# Assessing the effectiveness of an electrical stunning and chilling protocol for the slaughter of Atlantic mackerel (*Scomber scombrus*)

**DOI:** 10.1371/journal.pone.0222122

**Published:** 2019-09-04

**Authors:** Neil Anders, Bjørn Roth, Endre Grimsbø, Michael Breen

**Affiliations:** 1 Fish Capture Division, Institute of Marine Research (IMR), Bergen, Norway; 2 Department of Biological Sciences, University of Bergen, Bergen, Norway; 3 Nofima AS, Department of Processing Technology, Stavanger, Norway; 4 Marine Ecosystem Acoustics, Institute of Marine Research, Bergen, Norway; Radboud Universiteit, NETHERLANDS

## Abstract

Inducing unconsciousness in fish using electrical stunning prior to slaughter may improve fish welfare and fillet quality if such practises can be disseminated into wild capture fisheries. The objectives of this study were to: 1) evaluate if an established slaughter protocol consisting of dry electrical stunning (using a coupled AC/DC current at ≈ 110 V_rms_) followed by chilling could be used to stun the wild captured species Atlantic mackerel (*Scomber scombrus*) unconscious within 0.5 s; 2) determine if death could be induced without consciousness recovery by longer duration stunning (5 s) combined with chilling in an ice/water slurry for 6 min; and 3) examine the extent of quality defects arising from the applied slaughter protocol. We determined consciousness by examination of behavioural responses in a standardised vitality assessment. Out of a sample of 10 mackerel, 9 were assumed to be rendered unconscious by the 0.5 s stun, as determined by the presence of tonic and/or clonic muscle cramping consistent with a general epileptic insult. Assumed unconsciousness was maintained throughout chilling treatment in all fish (n = 25) following a full stun of 5 s. All fish were assumed to have died as a result of the protocol. There was no evidence of spinal damage or haematoma quality defects post filleting. These results suggest that the examined protocol is effective at slaughtering mackerel in a manner consistent with good welfare and without inducing quality defects, but further research is required to verify the unconscious condition via electroencephalogram (EEG) and before the procedure can be applied in wild capture fisheries.

## Introduction

Although welfare recommendations for farmed fish indicate that animals should be rapidly rendered insensible prior to death [1], there is currently no specific legislation governing slaughter practises in commercial wild capture fisheries. With a few exceptions, fish caught by pelagic trawl or purse seine are typically pumped aboard the catching vessel and chilled whole in either refrigerated seawater tanks or ice slurries for further processing ashore. In such situations, fish may be conscious and death may be a prolonged event [2]. Such pre-slaughter stress is not only suboptimal from an animal welfare perspective, but has also been shown to initiate behavioural and physiological responses which may negatively impact upon resulting flesh quality [3–5]. Notably, the sale price of fish can depend on quality [6–8] and potentially upon welfare status prior to death [9]. Modification of slaughter practises in wild capture fisheries so that fish are rendered unconscious to minimise pre-death struggling may therefore bring about both ethical and economic benefits.

Electrical stunning has the potential to rapidly induce unconsciousness in fish. Consistent with welfare recommendations [1,10,11], it has been shown that Atlantic salmon (*Salmo salar*) [12] can be rendered unconscious using a stun of 0.5 s duration. Effective 1 s stun durations have also been validated for a diverse range of freshwater and marine species [13–22] including the small pelagic Atlantic herring (*Clupea harengus*) [23]. Stunning efficiency is dependent on voltage and frequency [24,25], while impedance is independent of fish size [26]. The passage of sufficient electrical current through the head results in a general epileptiform insult due to depolarization of brain membrane potentials [27], during which the animal is unable to respond to stimuli and is assumed to be unconscious [28]. Undesirable rapid recovery of consciousness can however occur following such brief exposures to electrical current. Recent studies have shown that the unconscious condition can be extended by additional or longer duration stuns [12,13,20,22].

The European Food Safety Authority (EFSA) recommends that confirmation of an unconscious condition post stunning is established using neurological measures of brain electrical activity such as electroencephalogram (EEG) ([29]). However, the use of EEG can be technically challenging [30]. Behavioural and reflex indications may provide a robust and easily obtainable alternative to EEG [29,30] but because they do not directly quantify neurological activity, the isolated use of such indicators give only an indication of the likely state of consciousness.

To ensure good welfare, death must be induced prior to consciousness recovery [1,10]. A variety of post electrical stunning slaughter methods have been investigated to date [2,12,17,20,22], including chilling by the use of cold water/ice slurries. This technique has been shown to effective at both inhibiting recovery and inducing death for a range of farmed species [13,16,20,31–33] and is analogous to the practise of chilling fish during onboard storage in the pelagic wild capture industry.

Despite the apparent effectiveness of the technique, electrical stunning in fish has previously been associated with physical quality defects ([34] and references therein). These defects include fractures or breakages to the spinal column, as well as haematomas along the spine and inside the flesh resulting from severe muscle contraction due to electrical stimulation of the neuromuscular system. For herring, 60% of electrically stunned fish had broken spines, making processing by automated filleting machines impracticable [23]. However, more recent work has shown that such quality defects can be minimised while still inducing an effective unconscious condition, if fish are dry stunned using a coupled AC/DC current with an additional high frequency component [12,32,35]. Nevertheless, susceptibility to stunning induced defects could be species specific due to differences in spinal column strength [23] and energetic capacity [36].

Here we report on an experiment to determine if an established protocol typically utilised for farmed fish slaughter is effective for the wild capture species Atlantic mackerel (*Scomber scombrus*). Mackerel supports extensive fisheries [37] and establishing an effective slaughter method for this species may facilitate widespread fish welfare improvements in pelagic wild capture fisheries. The protocol consisted of dry electrical stunning followed by chilling. The objectives were to: 1) determine if mackerel could be rendered unconscious immediately (i.e. within 0.5 s) using dry electrical stunning; 2) establish if additional stunning would render mackerel unconscious without recovery for sufficient duration for death to occur during chilling; and 3) examine the extent of quality defects arising from the applied slaughter protocol.

## Materials and methods

### Fish capture

Wild mackerel were passively attracted using aquaculture feed pellets into a 12 x 12 x 12m aquaculture sea cage at the Austevoll Aquaculture Research Station (60°N, 005°E) of the Institute of Marine Research, Norway during the summer and autumn of 2018. Retained fish foraged on natural sources of food that washed into the pen with the current. The experiment was conducted on the 13^th^ and 14^th^ November 2018 (sea temperature at 0.5m depth of 10.6 and 10.7°C respectively). Fish were first removed from the net pen individually using barbless handlines and transported to the stunner in ~90L seawater buckets lined with plastic bags. Typically, < 10 min elapsed between capture to stunning. Mean (± SD) fish size used in the experiment was 37 ± 2 cm (fork length) and 603 ± 112 g; similar in size to fish targeted by the commercial fleet [38].

### Electrical stunner and settings

**Fig 1:**
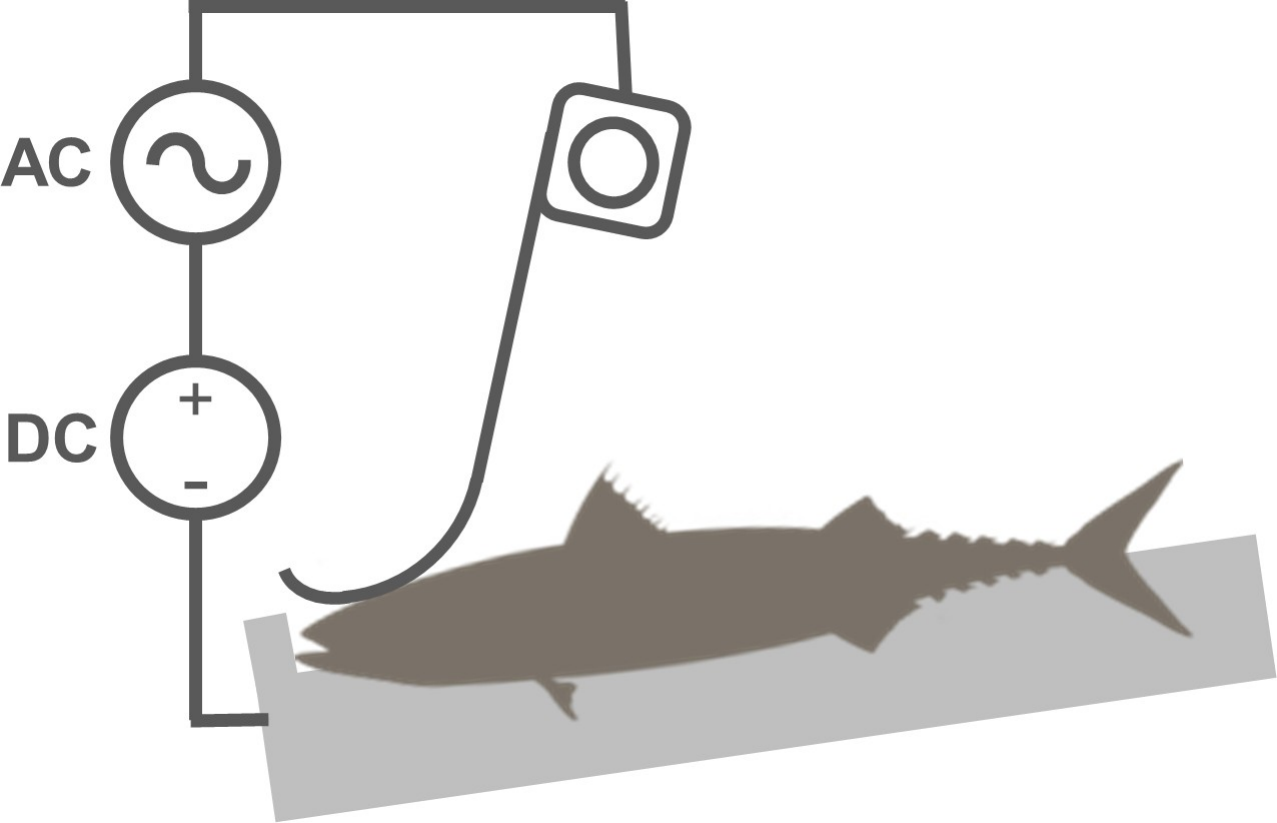
Electrical stunner schematic. Schematic of the dry electrical stunner and coupled AC/DC supply used to stun Atlantic mackerel. Note that fish were placed laterally into the stunner. Figure adapted from Fig 2 in [12].

We employed a commercial electrical dry stunner (STANSAS, Seaside A/S, Stranda, Norway) in combination with a time relay to control current duration. The stunner consisted of a metal base plate functioning as one electrode, with a hinged metal “paddle” hanging above (Fig 1) as the second electrode. During stunning, fish were exposed to a combined AC/DC supply (≈ 110V_rms_). The stunning current was dominated by the DC signal but with a non-sinusoidal AC component. Further detail regarding electrical current characteristics can be found in [12]. We measured voltage and current duration of stunning and the induced current (amperage) through individual fish using a portable oscilloscope (Fluke ScopeMeter 123) with a AC/DC current probe (Fluke 80i-110s, www.fluke.com).

### Experimental procedure

We first verified that mackerel (n = 10) could be rendered unconscious rapidly using an electrical stun of 0.5 s duration. One fish at the time was taken by hand and placed laterally into the electro-stunner, with the hanging electrode touching the upper side of the head and the metal base plate making contact with the whole of the underside of the body (Fig 1). A 0.5 s duration stun was then applied. We assumed the occurrence of either tonic and/or clonic phase muscle cramping post-stunning indicated a general epileptic insult and therefore unconsciousness [28].

We then determined if death could be induced prior to consciousness recovery, by application of a longer duration electrical stun followed by chilling. Before the cessation of any epileptic cramping, previously stunned fish were reintroduced into the stunner and exposed for a further 4.5 s. The remaining fish used in the experiment (n = 15) were exposed to an initial stun of 5 s with no secondary stun; the presence or absence of tonic and/or clonic phase muscle cramping post-stunning was also noted for these fish. Therefore, all fish used in the experiment (n = 25) were exposed to electrical stunning of 5 s total duration. For the animals exposed to an initial stun of 5 s, we measured the electrical current (in amps, A) passing through individual fish.

As soon as possible following complete stunning, fish were placed in a shallow freshwater/ice slurry bath. Mean (± SD) temperature of the bath was -0.69 ± 0.4°C (~11°C colder than ambient seawater temperatures on the day) with dissolved oxygen content of 10.38 ± 0.08 mg/L (≈ 88.9 ± 0.7% air saturation) as measured by an OxyGuard Handy Polaris oxygen meter (www.oxyguard.dk). We determined the state of consciousness by evaluating behaviour and reflex responses in individual fish once per minute for a total of 6 minutes post-stunning after removing the fish from the bath by hand. Three consciousness indicators (vestibular ocular reflex, rhythmic opercular activity and response to tactile stimuli) were assessed and assigned a score based on the strength of the response (Table 1, based on the protocols described by [30]). The time to assess the indicators was typically < 15 s, after which the fish was placed back into the bath. At 6 min post-stunning, the status of fish was decided. Consistent indicators of consciousness throughout the assessment period indicated the fish was able to recover and should be euthanised; otherwise fish were assumed to have died and were placed into a secondary water/ice slurry box. To further ensure that recovery did not occur, additional intermittent observations of consciousness were undertaken on these fish for the next ≈ 30 mins using the indicators described in Table 1

**Table 1:** Mackerel consciousness indicators. Indicators and associated procedures used to determine consciousness state in mackerel. Based on the protocols described by [30].

Indicator	Type	Procedure	Score	Description
Vestibulo-ocular reflex (VOR)	Reflex response	Rotate the fish around anterior-posterior axis	0	Eyes fixed relative to head
			1	Partial VOR or one eye shows VOR
			2	Eyes roll relative to the head while attempting to remain upright
Rhythmic opercular activity	Reflex response	Observe opercula for rhythmic movement (discount sporadic operculum flaring)	0	No opercula movement
			1	Slow or irregular movement
			2	Regular opercula movement
Response to tactile stimuli	Stimuli response	Administer a sharp pinch by hand in caudal peduncle area	0	No response
			1	Slow or feeble response
			2	Immediate vigorous escape attempt on first pinch

As recommended by [39], we confirmed that the indicators we employed were appropriate for mackerel by examining the same consciousness indicators (Table 1) but in a separate group of non-electrically stunned animals (n = 5). For this, mackerel which had been housed in a 3m diameter tank for 8–10 weeks were caught and removed by dipnet. Their state of consciousness was then assessed in the same way as for the main experiment (Table 1), prior to the fish being euthanised for use in other experimental procedures (not reported here).

Following stunning, fish were stored on ice for either ~18–24 hours (n = 22) or ~1 hour (n = 3) prior to filleting by hand. The presence or absence of the typical electrical stun induced quality defects [23] of haematomas (inside of the muscle and/or along the spine) and spinal column fractures or breakages was then recorded.

### Data analysis

We calculated a consciousness index for individual fish at each assessment time as follows: the sum of scores / the total possible score of 6. We assumed a consciousness index score of 0 indicated deep unconsciousness and that a score of 1 indicated fully conscious. We examined mean consciousness scores over time in association with bootstrap generated (repetitions = 10000) percentile 95% confidence intervals from the “boot” package [40] for R (version 3.4.2 [41]).

### Ethics statement

All experimental procedures were prospectively approved by the Norwegian animal welfare authority (Mattilsynet, FOTS licence ID: 15113) and were conducted at the Austevoll Aquaculture Research Station (60°N, 005°E) of the Institute of Marine Research, Norway. All procedures were undertaken by researchers with FELASA (Federation of European Laboratory Animal Science Associations) accredited laboratory animal science training. Prior to experimentation, fish were housed with conspecifics to ensure behavioural enrichment. Experimental design considered the 3R’s (Replacement, Reduction and Refinement). There was no practical alternative to the use of live animals. The total number of animals used (n = 25) was the minimum required to obtain a reasonable threshold of certainty for statistical validation. Minimisation of suffering and distress was inherent to the experimental design in that the objective was to render the fish unconscious by electrical stunning prior to inducing death. No anaesthesia was used. To ensure welfare, behaviour of experimental subjects was monitored for indications of consciousness recovery once per min following stunning. The pre-defined humane endpoint was consistent behavioural indications of consciousness recovery post stunning. In such cases, fish were to be euthanised immediately using a percussive blow to head; a legal and humane killing method for fish [42]. No fish had to be euthanised; all were killed by the combination of electrical stunning and the chilling treatment while unconscious. The exposure of conscious subjects to experimental treatment did not exceed 5 s; while unconscious the exposure for individual animals did not exceed 10 minutes.

## Results

In reference to the consciousness index we calculated, all non-stunned mackerel had a score of 1 and were classified as fully conscious (Fig 1) validating the indicators we examined as appropriate. Exposure to a single stun of 0.5 s at ≈110V_rms_ resulted in 90% (n = 9 out 10) of mackerel exhibiting tonic/clonic muscle cramping which is indicative of a general epileptic insult and were therefore assumed to be fully unconscious. The one fish which did not respond in this way was incorrectly positioned during stunning due to the animal moving immediately prior to the application of the current. All other fish (n = 15) displayed epileptic muscle cramping following application of the 5 s stun. Mean (±SD) induced current through individual fish (n = 13) was 0.68 ± 0.12 A.

**Fig 2:**
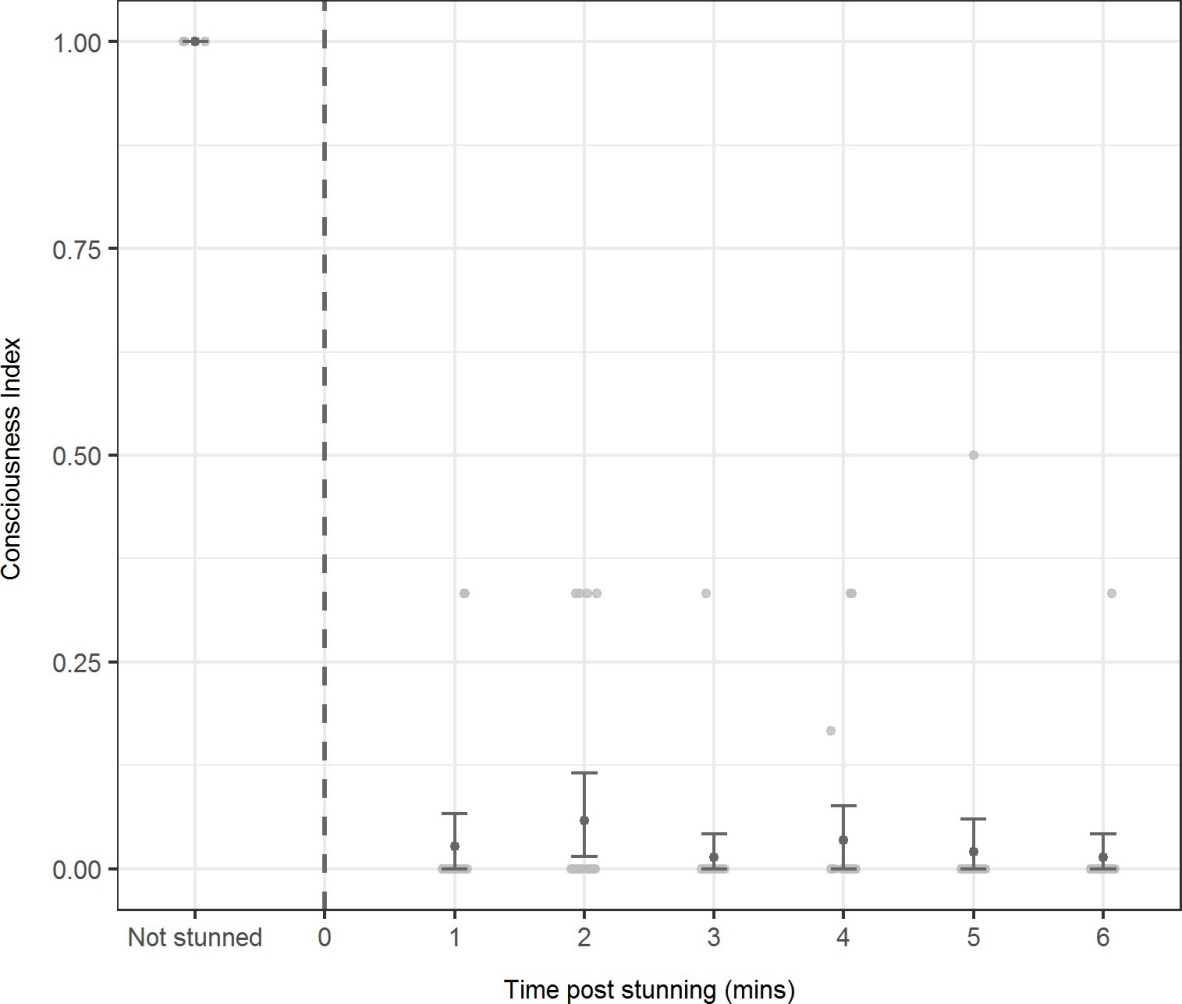
Consciousness index scores. Mean (±95% confidence interval) consciousness index scores for Atlantic mackerel, either not electrically stunned or post-stunning in combination with chilling. The grey vertical dotted line indicates the time at which the application of the 5 s electrical stun ceased. The underlying raw data is shown as grey points and has been horizontally jittered to reduce the incidence of overlapping datapoints.

The mean consciousness index scores post-stunning (Fig 2) indicated that the assumed unconscious condition continued during the chilling treatment and that all fish were assumed to be effectively non-conscious throughout. Overall mean (±SD) consciousness score post stunning was 0.03 ± 0.09. Considering the upper bound of the bootstrapped 95% confidence intervals, the true overall mean score post stunning could be as high as 0.07. At no time was any animal considered fully conscious.

Although a few animals showed indications of some degree of consciousness recovery (that is, index scores ≠ 0) during some assessments (Fig 2), these were rare (occurring in only 8% of all 145 assessments undertaken) and inconsistent events (in only 2.5% of consecutive assessments was the same fish assigned an index score ≠ 0). The highest post-stunning consciousness score was one assessment of 0.5. One animal exhibited an index score of ≠ 0 at the 6 min assessment time. For this case, we also assessed consciousness at 7 and 10 min post stunning. Index scores of 0 were obtained for both of these additional time points.

Following completion of the protocol, all animals were assumed to be dead. This assessment was supported by there being no indication of any recovery in any animal for the next ≈ 30 mins once placed in the secondary ice bath.

There was no evidence of spinal damage or blood haematomas in any fish post-filleting.

## Discussion

Previous work has shown the protocol we applied can successfully and rapidly render a variety of farmed fish species unconscious, that longer duration stunning combined with chilling can induce death prior to consciousness recovery [13,20,22,32,33] and that application of a combined AC/DC current results in low incidence of stunning related quality defects [12,35]. In accordance with these studies, our results indicate that the protocol can be successfully applied with the same outcomes (assuming that a lack of behavioural indicators is indicative of unconsciousness) on a commercially exploited wild caught species such as Atlantic mackerel.

The induction of a general epileptic insult (and therefore presumably unconsciousness) as indicated by the presence of tonic/clonic muscle cramping in the majority of our mackerel post stunning is consistent with findings for other species [12–22] and current understanding of the effect of electrical current on brain activity [12,27,28]. Post-stunning rhythmic operculum activity was absent for most of our mackerel and electrical stuns have been shown to result in irregular heart fibrillation [12]. Furthermore, rapid temperature reduction has been shown to reduce ventilation and cardiac output in fish [43]. Taken together with the potential for hypothermia to reduce brain metabolic rate and nerve action potential [32], it seems reasonable to assume that the chilling treatment we applied both prolonged the assumed unconscious condition initiated by electrical stunning, as well as causing some degree of functional hypoxia. Our assumption that all fish died while still unconscious is therefore reasonable, especially considering the oxyphilic nature of mackerel [44]. The relatively small size of mackerel (meaning heat loss can be expected to be high [45]) may make the protocol we applied particularly effective for this species.

From a commercial perspective, it is encouraging that no mackerel displayed signs of stunning induced quality defects. Previous stunning work on herring [23], another small wild caught pelagic species, resulted in high incidence of spinal injuries and hematomas. Our use of a combined AC/DC current with a high frequency component may have helped to avoid such issues by reducing the strength of muscle contractions [32]. Also, the spinal column of mackerel is more substantial compared to herring and therefore less likely to break. The strength of muscular contractile force during electrical stunning is related to the energetic status of the tissue [36]. The mackerel we used were probably not fully rested due to the stress of capture and transport to the stunner and therefore contractions during stunning were probably less than their full potential. This effect may have also influenced the low incidence of spinal damage, but is analogous to a wild capture situation where fatigue is highly likely during the capture process in purse seines and pelagic trawls [46].

Developing an efficient method for pumping typically large pelagic catches (normally in the region of hundreds of tonnes) from nets in combination with 5 s electrical stunning will be challenging. If additional welfare issues are to be avoided, pumping should be accomplished rapidly to reduce prolonged exposure of capture related stressors inside the net. Our results should therefore be considered as a first indication that it is possible to slaughter mackerel using the examined protocol, but further work along with possibly new technology is required before it can be implemented by the industry. This said however, the most practical location for the application of dry stunning onboard a commercial fishing vessel is likely post-pumping when the fish pass over the metal base plate of the dewatering unit. The plate could act as an electrode with multiple hanging electrodes suspended above as in the equipment used in this study. To ensure the fish are stunned in “dry” conditions, modifications to dewatering units currently in use may be required. The one fish in our study that did not display a clear epileptic response was incorrectly positioned in the stunner due to the animal moving prior to the application of the stun. This likely explains the lack of epileptic response in this animal but highlights the likely challenge of consistently stunning individual animals during high volume commercial pumping operations at sea.

Before any dissemination into commercial practise, the work detailed in this paper should certainly be expanded and upscaled. Of particular importance is experimentally confirming by EEG that the behavioural/reflex indicators we used are an accurate method of determining mackerel consciousness/death. This is especially important due to the previously noted lack of complete concordance between EEG and behavioural/reflex indicators [12] and for adherence to best practice principles [29]. It is also relevant to note that the content and distribution of fat in individual mackerel is highly seasonal [46,47] and that fat is a relatively good insulator of electrical current [47,48]. Consequently, the effectiveness of the protocol we examined may potentially depend upon season, but we were unable to test for this. Optimisation of exposure time may indicate that a 5 s stun duration is longer than required to ensure unrecoverable unconsciousness for this species, which would make at-sea stunning during high volume pumping more feasible. Future work should also focus upon the post-mortem flesh quality implications associated with the depletion of energy reserves arising from electrical stunning [48,49]. Determination of the effectiveness of stunning in non-“head first” orientations [13] should also be examined, as such orientations are likely to arise if stunning is to be conducted during dynamic at-sea pumping operations. Monitoring temperatures used to chill fish onboard commercial vessels could also be informative, to verify the chilling treatment we applied is representative of commercial situations.

## Conclusions

The results suggest that mackerel can be rendered unconscious within 0.5 s of application of an electrical current consisting of a coupled AC/DC current at ≈110V_rms_. Furthermore, they infer that mackerel do not recover consciousness and that death can be induced following application of a 5 s stun and subsequent chilling treatment. The examined protocol and electrical stunning signal do not have negative product quality implications with regards to spinal damage or haematomas. Further investigation using EEGs are is required to determine if the absence of the behavioural indicators we examined truly correspond to a state of unconsciousness/death in mackerel. Further practical development of the protocol is also required before it can be disseminated into commercial practice.

## Supporting information

S1 Dataset Consciousness index scores. Consciousness indicator scores and consciousness index results for mackerel used in the experiment.
(CSV)

S2 Dataset Biometric, epileptic response and environmental data. Biometric data and epileptic response for individual mackerel used in the experiment. Also includes environmental conditions (temperature and oxygen content) of the chilling bath.
(CSV)

S1 Analysis R script for analysis and plotting. Contains the R script for the data analysis and production of figures.
(R) Click here for additional data file.
